# Dipeptidyl Peptidase-IV Inhibitory Activity and Related Molecular Mechanism of Bovine α-Lactalbumin-Derived Peptides

**DOI:** 10.3390/molecules25133009

**Published:** 2020-06-30

**Authors:** Jing Gao, Han Gong, Xueying Mao

**Affiliations:** 1 Beijing Advanced Innovation Center for Food Nutrition and Human Health, College of Food Science & Nutritional Engineering, China Agricultural University, Beijing 100083, China; gaokeke1992@126.com; 2 Key Laboratory of Functional Dairy, College of Food Science and Nutritional Engineering, China Agricultural University, Beijing 100083, China; gonghan@cau.edu.cn

**Keywords:** bovine α-lactalbumin hydrolysate, dipeptidyl peptidase-IV inhibition, bioactive peptide, molecular docking

## Abstract

Identifying DPP-IV inhibitory peptides from dietary protein has attracted increased attention. In the present study, bovine α-lactalbumin hydrolysates were generated by alcalase for various hydrolysis times, and DPP-IV inhibitory activity of these hydrolysates was determined. The 4 h hydrolysates displayed the most potent DPP-IV inhibitory activity, with DPP-IV inhibition rate of 82.30 ± 1.39% at concentration of 1.0 mg/mL. DPP-IV inhibitory peptides were isolated from the 4 h-hydrolysates with gel filtration chromatography and reversed-phase high-performance liquid chromatography (RP-HPLC). Using liquid chromatography-electrospray ionization tandem mass spectrometry (LC-ESI MS/MS), two DPP-IV inhibitory peptides were identified, and their amino acid sequences were Glu-Leu-Lys-Asp-Leu-Lys-Gly-Tyr (ELKDLKGY) and Ile-Leu-Asp-Lys-Val-Gly-Ile-Asn-Tyr (ILDKVGINY), respectively. Furthermore, molecular docking analysis showed that peptides ELKDLKGY and ILDKVGINY could form hydrogen bonds, pi-cation interactions, and salt bridges with DPP-IV. These findings indicated that bovine α-lactalbumin may be a potential source of natural DPP-IV inhibitor.

## 1. Introduction

Diabetes, which is a worldwide chronic disease, affects 463 million people in 2019, and this number is expected to reach 700 million by 2045 [1]. Type 2 diabetes is the most prevalent form, accounting for approximately 90% of cases [1]. Type 2 diabetes is characterized by insufficient pancreatic insulin secretion and/or insulin resistance in peripheral tissues, and is associated with various complications such as retinopathy, nephropathy and neuropathy, thus resulting in serious harm to human health [2,3].

Recently, dipeptidyl-peptidase IV (DPP-IV) inhibitors have become a novel therapeutic approach to manage type 2 diabetes [4]. DPP-IV is a ubiquitous enzyme widely known for degradation of glucagon-like peptide-1 (GLP-1) and glucose-dependent insulinotropic polypeptide (GIP), which contribute to 50–60% of insulin secretion in a glucose-dependent way [5,6]. Therefore, inhibition of DPP-IV could maintain the insulinotropic activity of GLP-1 and GIP, resulting in improved glucose homeostasis in type 2 diabetes [7]. There are several synthetic DPP-IV inhibitors in use, e.g., sitagliptin, vildagliptin, saxagliptin, linagliptin, and alogliptin, which have been demonstrated to display protective effects against type 2 diabetes [6,8,9,10,11]. Despite possessing a high hypoglycemic activity, these DPP-IV inhibitors have shown some adverse side effects, such as asthenia, headache, nasopharyngitis, upper respiratory tract infections, cardiac and vascular issues [12,13,14]. Therefore, searching for novel DPP-IV inhibitors as alternative options has attracted increased attention.

Dietary protein-generated hydrolysates and peptides have been demonstrated to possess DPP-IV inhibitory activity in silico, in vitro, and in vivo. For example, 294 edible proteins and 5 commercial proteases were subjected to in silico analysis, the result indicated that they contain numerous potential DPP-IV inhibitory peptides [15]. In vitro, camel skin gelatin hydrolysates were demonstrated to display highly potent DPP-IV inhibitory activity [16]. In vivo, yam dioscorin-derived peptide RRDY reduced blood DPP-IV activity and increased blood insulin levels in normal mice, resulting in decreased postprandial blood glucose [17].

Bovine whey is an important by-product of cheese industry, which has high pollution potential [18]. Bovine α-lactalbumin is the second most abundant protein in whey, accounting for approximately 17% of whey protein [19]. Therefore, the utilization of α-lactalbumin makes great sense in the governance of environmental problems caused by whey by-products. Previous in silico studies have shown that bovine α-lactalbumin contain DPP-IV inhibitory peptide sequences, which was confirmed by the potent DPP-IV inhibitory activity of bovine α-lactalbumin hydrolysates and bovine α-lactalbumin derived peptides [20,21,22,23,24]. However, the underlying molecular mechanism of α-lactalbumin derived peptides on DPP-IV inhibition has not been understood clearly. In our previous studies, we have demonstrated that bovine α-lactalbumin hydrolysates prepared by alcalase attenuated adipose insulin resistance and nonalcoholic fatty liver disease in mice fed with high-fat diet [25,26]. Due to the connection between glycolipid metabolism-regulating activity and DPP-IV inhibitory activity [27,28], we hypothesized that bovine α-lactalbumin hydrolysates obtained by alcalase possessed DPP-IV inhibitory activity.

Thus, in the present study, bovine α-lactalbumin was hydrolyzed by alcalase to prepare hydrolysates, and the DPP-IV inhibitory activity was determined. The hydrolysates with the most potent DPP-IV inhibitory activity were purified by gel filtration chromatography and reversed-phase high-performance liquid chromatography (RP-HPLC), and liquid chromatography-electrospray ionization tandem mass spectrometry (LC-ESI MS/MS) was used to identify DPP-IV inhibitory peptides. In the last, molecular docking analysis was used to investigate the interaction between DPP-IV and the identified peptides.

## 2. Results

### 2.1. Degree of Hydrolysis and DPP-IV Inhibitory Activity of Bovine α-Lactalbumin Hydrolysates

**Figure 1:**
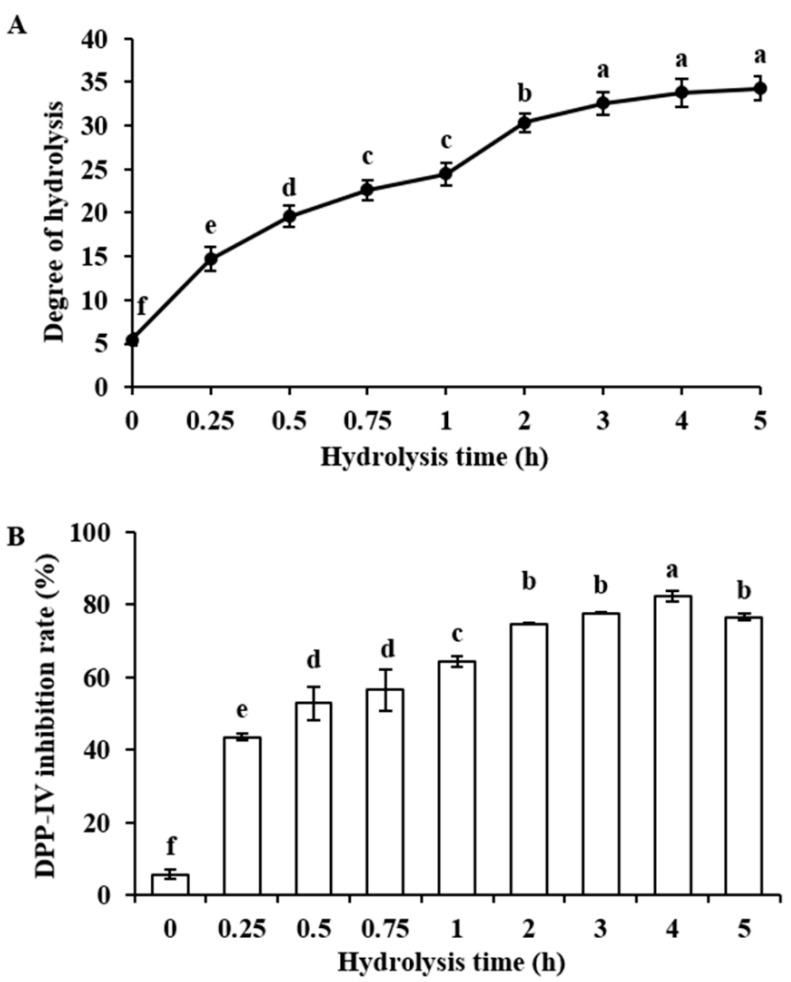
Degree of hydrolysis (DH) (**A**) and Dipeptidyl peptidase-IV (DPP-IV) inhibitory activity (**B**) of bovine α-lactalbumin hydrolysates. Bovine α-lactalbumin was hydrolyzed with alcalase (enzyme/substrate ratio = 5%, 0–5 h). The DPP-IV inhibition rate was measured using 1.0 mg/mL of hydrolysate (final assay concentration, protein basis). Values are expressed as means ± SD of 3 independent determinations. Different letters indicate significant differences (*p* < 0.05).

Bovine α-lactalbumin was hydrolyzed by alcalase for 0, 0.25, 0.5, 0.75, 1, 2, 3, 4, and 5 h, and nine hydrolysates were obtained. Degree of hydrolysis (DH) of the obtained hydrolysates increased as hydrolysis time prolonged from 0 h to 3 h, and there was no significant difference between DH of hydrolysates obtained at 3 h, 4 h, and 5 h (Figure 1A). DPP-IV inhibitory activity of bovine α-lactalbumin hydrolyzed by alcalase at a concentration of 1.0 mg/mL was shown in Figure 1B. Compared to non-hydrolyzed bovine α-lactalbumin with DPP-IV inhibition rate of 5.74 ± 1.31%, the hydrolysates prepared by alcalase at each hydrolysis time displayed more potent DPP-IV inhibitory activity (with DPP-IV inhibition rate from 43.53 ± 0.77% to 82.30 ± 1.39%) (*p* < 0.05). The DPP-IV inhibition rate of hydrolysates enhanced as hydrolysis time increased from 0 h to 4 h, and reached the maximum value at 4 h (82.30 ± 1.39%). However, with the elongation of hydrolysis time increased to 5 h, the DPP-IV inhibition rate of the obtained hydrolysates was 76.47 ± 0.95%, which was not significantly higher than that of 4 h hydrolysates (*p* < 0.05). Thus, the 4 h hydrolysates were selected for further analysis.

### 2.2. DPP-IV Inhibitory Activity of Bovine α-Lactalbumin Hydrolysates Fractionated by Sephadex G-25 Gel Filtration Chromatography

**Figure 2:**
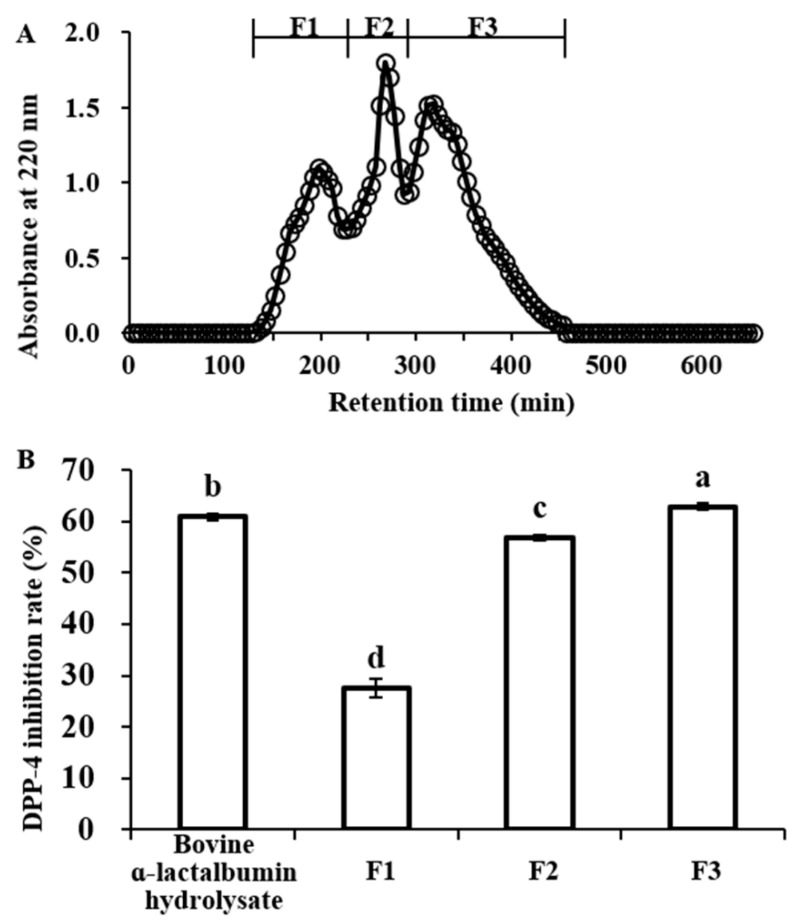
Elution profile (**A**) and dipeptidyl peptidase-IV (DPP-IV) inhibitory activity (**B**) of bovine α-lactalbumin hydrolysate fractions obtained by Sephadex G-25 gel filtration chromatography. The DPP-IV inhibition rate was determined using samples at concentration of 0.5 mg/mL (final assay concentration). Values are expressed as means ± SD of 3 independent determinations. Different letters indicate significant differences (*p* < 0.05).

The obtained bovine α-lactalbumin hydrolysates with the highest DPP-IV inhibitory activity were separated by Sephadex G-25 gel filtration chromatography, and three individual fractions (F1, F2, F3) were obtained, as shown in Figure 2A. DPP-IV inhibitory activity of bovine α-lactalbumin hydrolysates and each fraction was determined at a concentration of 0.5 mg/mL. Compared with un-separated α-hydrolysates whose DPP-IV inhibition rate was 60.90 ± 0.54%), F1 and F2 displayed weaker DPP-IV inhibitory activity (with DPP-IV inhibition rate of 27.53 ± 1.77% and 56.92 ± 0.54%, respectively). However, the F3 fraction possessed more potent DPP-IV inhibitory activity than un-separated α-hydrolysates (with DPP-IV inhibition rate of 62.88 ± 0.61%, *p* < 0.05) (Figure 2B). Thus, the F3 fraction was performed for further purification by RP-HPLC.

### 2.3. DPP-IV Inhibitory Activity of F3 Fractionated by RP-HPLC

**Figure 3:**
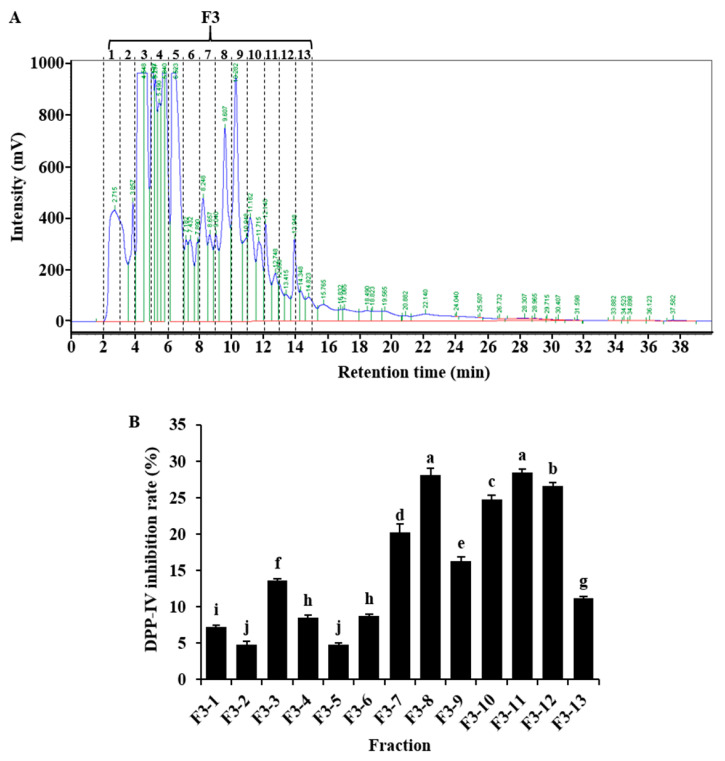
High-performance liquid chromatography profile of F3 fraction from Sephadex G-25 gel filtration chromatography (**A**) and dipeptidyl peptidase-IV (DPP-IV) inhibitory activity of fractions (**B**). The DPP-IV inhibition rate was determined using sample of 0.024 mg/mL (final assay concentration). Values are expressed as means ± SD of 3 independent determinations. Different letters indicate significant differences (*p* < 0.05).

To further separate the highest DPP-IV inhibitory fraction, RP-HPLC was used to purify F3 fraction obtained by Sephadex G-25 gel filtration chromatography. As shown in Figure 3A, after 30 min elution, 13 major peaks were detected, based on which the fractions (numbered from F3-1 to F3-13) were collected. All the fractions were subjected to DPP-IV inhibitory activity determination at a concentration of 0.024 mg/mL (final assay concentration). Results showed that F3-8 and F3-11 fractions exhibited the highest DPP-IV inhibitory activity with values of 28.10 ± 0.41% and 28.40 ± 0.45%, respectively (Figure 3B).

### 2.4. Identification of DPP-IV Inhibitory Peptides

**Figure 4:**
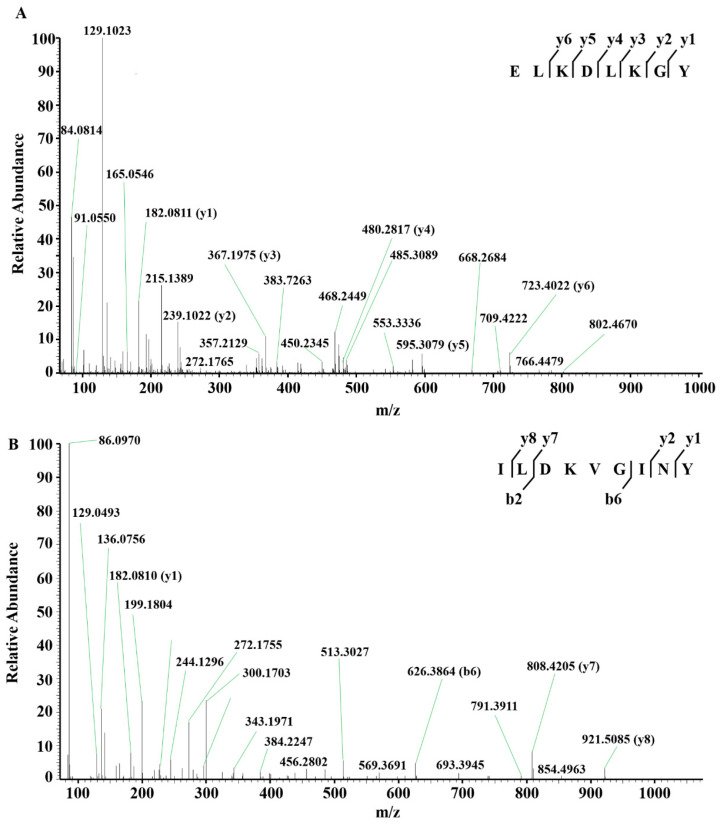
Identification of DPP-IV inhibitory peptides. (**A**) LC-MS/MS spectrum of single-charged ion with *m*/*z* 483.26996. Following sequence interpretation and database searching, the MS/MS spectrum of ion with *m*/*z* 483.26996 was matched to peptide ELKDLKGY. (**B**) LC-MS/MS spectrum of single-charged ion with *m*/*z* 517.79901. Following sequence interpretation and database searching, the MS/MS spectrum of ion with *m*/*z* 517.79901 was matched to peptide ILDKVGINY. Peptide sequences are displayed with ion fragments observed in the spectrum.

**Figure 5:**
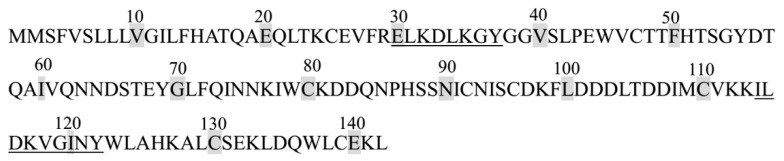
Mature amino acid sequences of bovine α-lactalbumin. Peptide sequences identified in the bovine α-lactalbumin fractions are underlined.

F3-8 and F3-11 fractions obtained by RP-HPLC were subjected to LC-MS/MS analysis for peptide sequence identification. As listed in Table 1, the peptide sequences of the F3-8 and F3-11 were Glu-Leu-Lys-Asp-Leu-Lys-Gly-Tyr (ELKDLKGY) and Ile-Leu-Asp-Lys-Val-Gly-Ile-Asn-Tyr (ILDKVGINY), respectively. Figure 4A showed the LC-MS/MS spectrum of single-charged ion with *m*/*z* 483.26996, which matched to sequence ELKDLKGY corresponding to bovine α-lactalbumin f (30–37). Figure 4B showed the LC-MS/MS spectrum of single-charged ion with *m*/*z* 517.79901, which matched to sequence ILDKVGINY corresponding to bovine α-lactalbumin f (114–122). Mature amino acid sequences of bovine α-lactalbumin are shown in Figure 5, and peptide sequences identified in F3-8 and F3-11 fractions are underlined.

### 2.5. Molecular Docking Analysis of Bovine α-Lactalbumin Derived Peptides to DPP-IV

**Figure 6:**
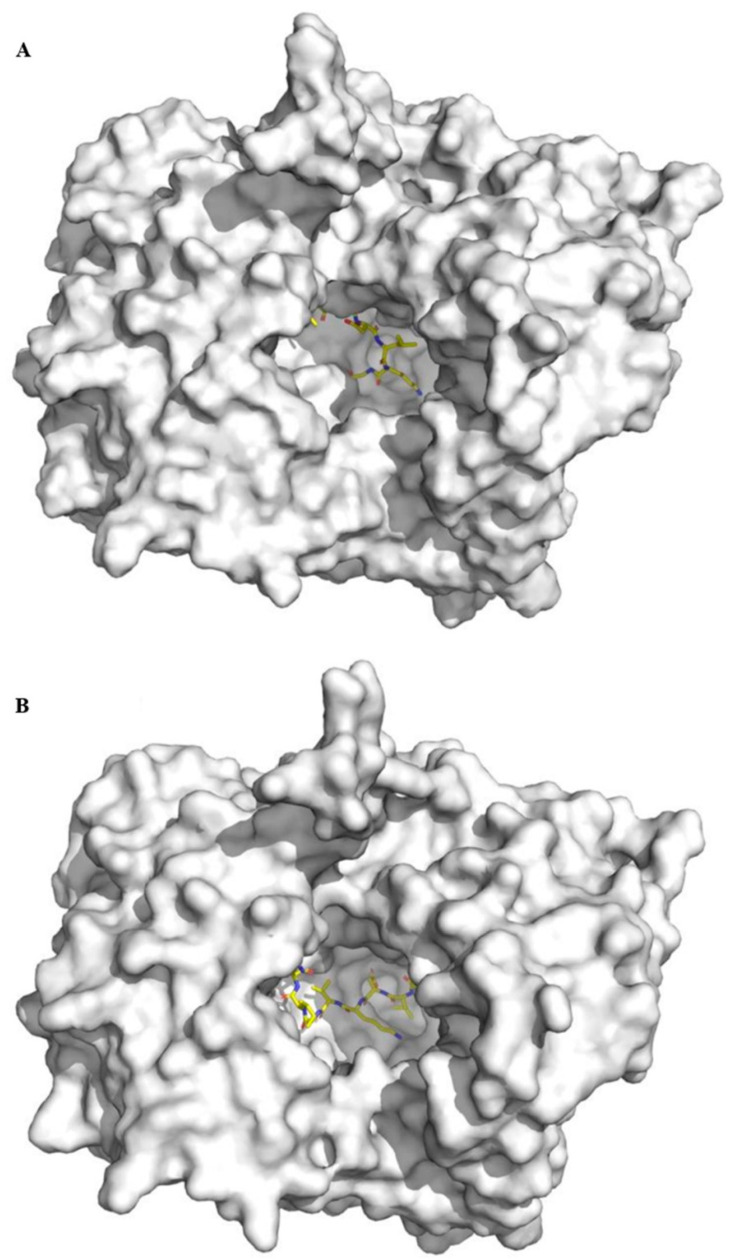
3D binding model of ELKDLKGY (**A**) and ILDKVGINY (**B**) with the target DPP-IV. The receptor (DPP-IV) is depicted as white and the ligand (peptide) is depicted as yellow.

**Figure 7:**
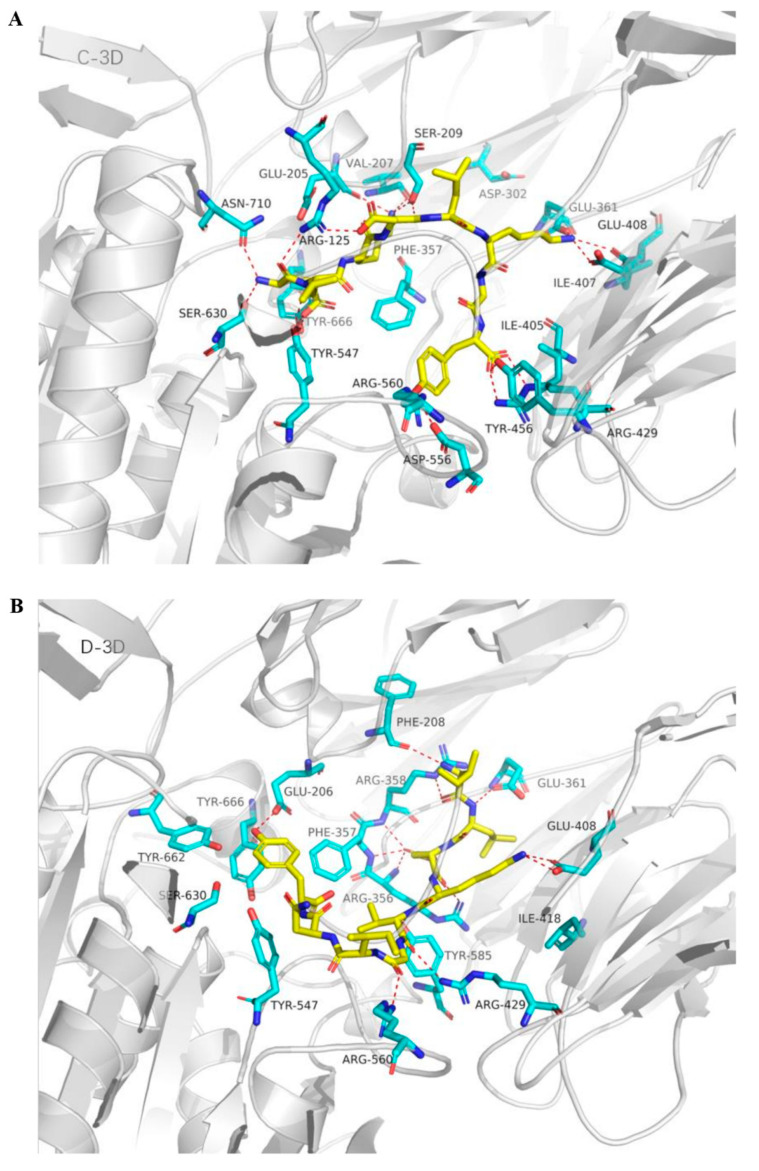
Details of ELKDLKGY (**A**) and ILDKVGINY (**B**) interaction at the DPP-IV interaction sites. Peptide ligands are colored in yellow, and the surrounding residues in the binding pockets are colored in cyan. The backbone of the receptor is depicted as light gray ribbon. The red dot line shows the hydrogen bond between ligand and receptor.

In order to investigate the interaction between DPP-IV inhibitory peptides and DPP-IV, molecular docking analysis was carried out. As shown in Table 2, docking score of the peptides ELKDLKGY and ILDKVGINY binding to target DPP-IV were −7.771 kcal/mol and −8.037 kcal/mol, respectively. ELKDLKGY was predicted to form fifteen hydrogen-bonds with Arg125, Ser630, Asn710, Tyr547, Ser209, Val207, Glu205, Glu361, Glu408, Arg429, Tyr456, and Asp556, four salt bridges with Arg125, Glu361, Glu408, and Arg429, and one pi-cation with Tyr666 (Table 3). ILDKVGINY was predicted to form six hydrogen-bonds with Arg356, Arg357, Arg358, Glu408, Arg429, and Arg560, two pi-cations with Arg358 and Arg125, and one salt bridge with Glu408. The 3D binding model of ELKDLKGY and ILDKVGINY with the target DPP-IV was shown in Figure 6 and Figure 7. The binding ability of peptides with the target DPP-IV was in the order of ILDKVGINY > ELKDLKGY.

## 3. Discussion

Glucagon-like peptide-1 (GLP-1) and glucose-dependent insulinotropic polypeptide (GIP) are incretin hormones that stimulate 50%−60% of insulin secretion in a glucose-dependent manner [29,30]. However, GLP-1 and GIP have a very short half-life (about 1−2 min) due to the degradation by dipeptidyl-peptidase IV (DPP-IV) [31,32]. Thus, DPP-IV inhibitors (such as sitagliptin, vildagliptin, saxagliptin, linagliptin, and alogliptin), which could inhibit the activity of DPP-IV, have become a novel anti-diabetic approach [29]. In the present study, bovine α-lactalbumin hydrolysates were obtained by alcalase, and the DPP-IV inhibitory activity was determined. Our results showed that 4 h hydrolysates possessed the strongest DPP-IV inhibitory activity with inhibition rate of 82.30 ± 1.39% at a concentration of 1.0 mg/mL (Figure 1), which was higher than that of bovine α-lactalbumin hydrolysate generated by elastase (with DPP-IV inhibition rate of 75.8 ± 3.7% at a concentration of 3.1 mg/mL) [24].

In order to verify the bioactive peptides, bovine α-lactalbumin hydrolysates were isolated with gel filtration chromatography and reversed-phase high-performance liquid chromatography (RP-HPLC), and identified with liquid chromatography-electrospray ionization tandem mass spectrometry (LC-ESI MS/MS). Among the two identified peptides shown in Table 1, peptide ILDKVGINY has been demonstrated with DPP-IV inhibitory activity previously (IC_50_ values = 263 μM) [22]. As for peptide ELKDLKGY, although it has not been mentioned in the literature previously, it contains the same amino acid sequences with several reported DPP-IV inhibitory peptides, such as RELKDLKGY, RELKDLKGYG, RELKDLKGYGG, RELKDLKGYGGVS, RELKDLKGY, KDL, KGY, and GY [22,24]. Various studies showed that the peptides displaying potent DPP-IV inhibitory activity generally have a length of 2 to 7 amino acids [30]. In the present study, octapeptide ELKDLKGY and nonapeptide ILDKVGINY are identified, which are very close to the length of DPP-IV inhibitory peptides reported previously. Previous studies also showed that the hydrophobic amino acids in peptides play an important role on their DPP-IV inhibitory activity [33]. Our results showed that the two DPP-IV inhibitory peptides derived from bovine α-lactalbumin hydrolysates were composed of several hydrophobic amino acids, including leucine (Leu, L), isoleucine (Ile, I), and valine (Val, V). This characterization may contribute to their DPP-IV inhibitory activities.

Finally, molecular docking analysis was performed to investigate the interaction mechanism between DPP-IV and DPP-IV inhibitors. Molecular docking analysis has been widely used to investigate molecular mechanism of DPP-IV inhibitory peptides [34,35]. The active site of DPP-IV contains two pockets as follows: a hydrophobic S1 pocket consisted of residues Tyr631, Val656, Trp659, Tyr662, Tyr666, and Val711; and a charged S2 pocket consisted of residues Arg125, Glu205, Glu206, Phe357, Ser209, and Arg358 [36]. In the present study, bovine α-lactalbumin derived peptide ELKDLKGY had interactions with Arg125, Arg429, Asn710, Asp556, Glu205, Glu361, Glu408, Ser209, Ser630, Tyr456, Tyr547, Tyr666, and Val207. Among these interactions, Tyr666 is located in the S1 pocket, and Arg125, Glu205, and Ser209 are located in the S2 pocket. ILDKVGINY could bind with residues Arg125, Arg356, Arg357, Arg358, Arg429, Arg560, and Glu408, among which Arg125 and Arg358 are locked in the S2 pocket. In molecular analysis, the lower score of free energy binding usually indicates the higher binding affinity between ligands and target DPP-IV [37]. Our present results showed that the docking scores of ELKDLKGY and ILDKVGINY are −7.771 kcal/mol and -8.037 kcal/mol, respectively, indicating that the binding ability with the target DPP-IV of ILDKVGINY is more potent than that of ELKDLKGY.

## 4. Materials and Methods

### 4.1. Materials

Bovine α-lactalbumin was provided by Davisco Foods International Inc. (Eden Prairie, MN, USA). Alcalase was purchased from Novozymes Biologicals Inc. (Bagsvaerd, Denmark). Gly-Pro-p-nitroanilide (H-Gly-Pro-pNA HCl) and dipeptidyl peptidase-IV (from porcine kidney) were purchased from Sigma-Aldrich (St. Louis, MO, USA). HPLC-grade acetonitrile and formic acid were purchased from Fisher Scientific (Fair Lawn, NJ, USA). All other chemicals used in this study were at least of analytical grade.

### 4.2. Preparation of Bovine α-Lactalbumin Hydrolysates

Bovine α-lactalbumin was dissolved in distilled water (5.0%, *w*/*v*), followed by preincubation at 85 °C for 15 min. The hydrolysis was initiated by adding alcalase at an enzyme/substrate mass radio of 1/20 at optimal conditions (pH 8.5, 55 °C). Hydrolysates of bovine α-lactalbumin were collected at 0, 0.25, 0.5, 0.75, 1, 2, 3, 4, 5 h. These hydrolysates were heated at 85 °C for 20 min to inactivate the enzyme, and centrifuged at 4000× *g* for 20 min at 4 °C to collect supernatants. After freeze-drying, the samples were stored at −20 °C for further investigation. Protein and peptide concentration was measured by bicinchoninic acid protein assay (BCA) kit (Tiangen Biotech, Beijing, China) according to the manufacture’s instruction. Briefly, 25 μL of sample was mixed with 200 μL of BCA working reagent (Reagent A to Reagent B ratio = 50:1), incubated at 37 °C for 30 min, and cooled to room temperature. Absorbance of the reaction mixture was measured at 562 nm, and protein concentration was calculated using bovine serum albumin (0–2 mg/mL) as a standard.

### 4.3. Determination of Degree of Hydrolysis

Trinitrobenzenesulfonic acid (TNBS) method was used to measure degree of hydrolysis as our previous study described [26]. Phosphate buffer (pH 8.2, 0.2125 M, 1 mL) and TNBS (0.1%, 1 mL) were added into bovine α-lactalbumin hydrolysate sample, incubated in dark at 50 °C for 60 min, and stopped by adding 0.1 M HCl (2 mL). The reaction mixture was cooled at room temperature for 30 min, and absorbance was measured at 420 nm with _L_-leucine as a standard. Degree of hydrolysis was calculated as (Equation (1)):

% Degree of hydrolysis = *h*/*h_tot_* × 100 (1)

*h*, the number of cleaved peptide bonds; *h_tot_*, the total number of peptide bonds per unit weight (for α-lactalbumin, *h_tot_* is 8.6 meq/g).

### 4.4. Determination of DPP-IV Inhibitory Activity

DPP-IV inhibitory activity was carried out using a method described previously with slight modifications [38]. Briefly, 20 μL of samples diluted in Tris-HCl buffer (100 mM, pH8.0) were pipetted onto a 96-well microplate, and mixed with 100 μL of H-Gly-Pro-pNA∙HCl (final concentration 0.5 mM). Then 30 μL of DPP-IV (final concentration 0.0075 U/mL) was added to initiate the reaction. The mixture was incubated at 37 °C for 30 min, and the absorbance was monitored at 405 nm. The DPP-IV inhibitory activity was calculated as follows (Equation (2)):

DPP-IV inhibition rate (%) = (1 − (Absorbance (test sample) − Absorbance (sample control))/(Absorbance (negative reaction) − Absorbance (negative control))) × 100(2)

where Absorbance (test sample) was the absorbance at 405 nm in the presence of DPP-IV, H-Gly-Pro-pNA∙HCl, and sample; Absorbance (sample control) was the absorbance at 405 nm when sample was replaced by Tris-HCl buffer (100 mM, pH 8.0); Absorbance (negative reaction) was the absorbance at 405 nm in the presence of DPP-IV and H-Gly-Pro-pNA∙HCl but with no sample; Absorbance (negative control) was the absorbance at 405 nm with no DPP-IV and sample.

### 4.5. Gel Filtration Chromatography

Bovine α-lactalbumin hydrolysates were separated with gel filtration chromatography according to the method described by Huang, Jao, Ho, and Hsu [39]. Freeze-dried hydrolysates (30 mg) were resuspended in deionized water and loaded onto a pre-equilibrated Sephadex G-25 column (2.5 cm × 70 cm, GE Healthcare Bio-Science AB, Beijing, China). The column was eluted with deionized water at a flow rate of 1.0 mL/min. The eluate was collected (5 mL) and the absorbance was measured at 220 nm. Fractions from at least 20 Sephadex G-25 runs were freeze-dried for further study.

### 4.6. Reversed-Phase High-Performance Liquid Chromatography (RP-HPLC)

Fraction obtained from gel filtration was further purified by RP-HPLC (model LC-10AT, Shimadzu, Kyoto, Japan) according to previously described methods with minor modifications [40,41]. Fraction dissolved in ultrapure water (1 mL) was loaded onto a RP-HPLC column (Eclipse XDB-C18, 4.6 mm × 250 mm, 5 μm, Agilent Technologies Inc., Shanghai, China) at a concentration of 2 mg/mL. Gradient elution was performed with ultrapure water (Mobile phase A) and acetonitrile (mobile phase B) by a linear gradient of B from 10% to 90% from 0 to 30 min at a flow rate of 1 mL/min at 30 °C, and the absorbance was monitored at 220 nm. Fractions eluting from 2 min to 16 min were collected and freeze-dried independently. In order to obtain enough samples for further analysis, the separation was carried out at least 10 times.

### 4.7. Identification of Peptide Sequence by Liquid Chromatography-Electrospray Ionization Tandem Mass Spectrometry (LC-ESI MS/MS)

Peptide identification was performed by LC-ESI MS/MS as previously described [42]. A nanoAcquity nano HPLC system (Waters, Milford, MA, USA) fitted with an Aqua C18 column (5 μm particle size, 125 Å, Phenomenex, Torrance, CA, USA) packed trap column (2 cm × 100 μm, Polymicro Technologies, Phoenix, AZ, USA) and an Aqua C18 column (3 μm particle size, 125 Å, Phenomenex, Torrance, CA, USA) packed microanalytical column (10 cm × 50 μm, Polymicro Technologies, Phoenix, AZ, USA) was used to separate peptides. Solvent A was 0.1% formic acid in water, and solvent B was 0.1% formic acid in acetonitrile. During the elution, the ratio of solvent B was increased from 1% to 40% (*v*/*v*) in 40 min at a flow rate of 200 nL/min. The HPLC system was coupled to a Q-Exactive high-resolution mass spectrometer (Thermo Scientific, Waltham, MA, USA). After separation with HPLC system, the peptide sample was directly injected into the MS system, and the analysis was performed under parameters as follows: data-dependent MS2 model, positive ion mode, MS scan range 100–1500 *m*/*z*, full scan resolution 70,000, MS/MS scan resolution 17,500. The MS/MS obtained data were preprocessed with Mascot Distiller 2.4 (Matrix Science, London, UK), and peptide sequences were determined by comparing with protein data of Bos taurus in UniProtKB/Swiss-Prot database using Mascot 2.4 search engine (Matrix Science, London, UK).

### 4.8. Molecular Docking

Molecular docking was used to determine the interaction between DPP-IV inhibitory peptides and DPP-IV. The crystal structures of DPP-IV were obtained from RCSB Protein Data Bank (PDB code 4PNZ). The “Protein Preparation Wizard” workflow in Maestrov (version 9.4, Schrödinger Inc., New York, NY, USA, 2015) was used to carry out the DPP-IV manipulation, which include removing water molecules, protonation, deal with bonds, loops and side chain reparation by Prime, and optimization based on OPLS_2005 force field. Moecular docking simulations for peptides and DPP-IV were performed by Peptide Docking in Schrödinger, and Glidescore for the Scoring method was used to score the peptide poses.

### 4.9. Statistical Analysis

All data were presented as means ± standard deviations (SD) values of triplicate determinations. One-way analysis of variance (ANOVA) followed by Duncan’s multiple range test (SPSS version 20.0, IBM Inc., Chicago, IL, USA, 2011) was used to evaluate significance between groups. *p* < 0.05 was considered statistically significant.

## 5. Conclusions

Nine bovine α-lactalbumin hydrolysates were generated by alcalase, among which 4-h hydrolysates displayed the most potent DPP-IV inhibitory activity. Two DPP-IV inhibitory peptides were isolated, their amino acid sequences were Glu-Leu-Lys-Asp-Leu-Lys-Gly-Tyr (ELKDLKGY) and Ile-Leu-Asp-Lys-Val-Gly-Ile-Asn-Tyr (ILDKVGINY). Molecular docking analysis showed that peptides ELKDLKGY and ILDKVGINY could bind with DPP-IV through hydrogen bonds, pi-cation interactions, and salt bridges. These results indicated that bovine α-lactalbumin can be considered as a potential source of natural DPP-IV inhibitor.

## Figures and Tables

**Table 1:** Liquid chromatography-electrospray ionization tandem mass spectrometry (MS) identification of dipeptidyl peptidase-IV (DPP-IV) inhibitory peptides in fraction F3-8 and F3-11 obtained by HPLC.

Sequence	Calculated Mass (Da)	Origin
**F3-8**		
ELKDLKGY	929.19	α-LA f (30–37)
**F3-11**		
ILDKVGINY	1016.77	α-LA f (114–122)

**Table 2:** Molecular docking score of peptides binding to target dipeptidyl peptidase-IV (DPP-IV).

Peptide (Ligand)	Target	Binding Energy Score (kcal/mol)
ELKDLKGY	DPP-IV	−7.771
ILDKVGINY	DPP-IV	−8.037

**Table 3:** Strong intermolecular contacts between the receptor (DPP-IV) and peptide.

Peptide	Peptide Residue	Receptor Residue	Type
ELKDLKGY	E1	Arg125	H-bond
	E1	Ser630	H-bond
	E1	Asn710	H-bond
	E1	Tyr547	H-bond
	E1	Tyr666	H-bond
	E1	Tyr666	Pi-cation
	K3	Ser209	H-bond
	K3	Val207	H-bond
	K3	Glu205	H-bond
	D4	Ser209	H-bond
	D4	Arg125	H-bond
	D4	Arg125	Salt bridge
	K6	Glu361	H-bond
	K6	Glu408	H-bond
	K6	Glu361	Salt bridge
	K6	Glu408	Salt bridge
	Y8	Arg429	H-bond
	Y8	Arg429	Salt bridge
	Y8	Tyr456	H-bond
	Y8	Asp556	H-bond
ILDKVGINY	I1	Arg358	Pi-cation
	D3	Arg356	H-bond
	D3	Arg357	H-bond
	D3	Arg358	H-bond
	K4	Glu408	H-bond
	K4	Glu408	Salt bridge
	V5	Arg429	H-bond
	G6	Arg560	H-bond
	Y9	Arg125	Pi-cation

## Notes

**Sample Availability:** Samples of the bovine α-lactalbumin are available from the authors.
